# Editorial: The cardiac stroma in homeostasis and disease

**DOI:** 10.3389/fcvm.2023.1248750

**Published:** 2023-07-10

**Authors:** Isotta Chimenti, Roberto Gaetani, Francesca Pagano

**Affiliations:** ^1^ Department of Medical Surgical Sciences and Biotechnologies, Sapienza University, Latina, Italy; ^2^ Mediterranea Cardiocentro, Napoli, Italy; ^3^ Department of Molecular Medicine, Sapienza University, Rome, Italy; ^4^ Department of Bioengineering, Sanford Consortium for Regenerative Medicine, University of California, San Diego, CA, United States; ^5^ Institute of Biochemistry and Cell Biology, National Council of Research (IBBC-CNR), Monterotondo, Italy

**Keywords:** cardiac stroma, cardiac fibroblasts, cardiac inflammation, cardiac remodelling, cardiac macrophages

**Editorial on the Research Topic** The cardiac stroma in homeostasis and disease

The healthy architecture of the cardiac tissue is maintained through a complex balance between the parenchymal cells, namely cardiomyocytes with contractile function, and the stromal counterpart which serves several fundamental homeostatic functions. These compartments exchange signals in both physiological and pathological conditions, in response to many stimuli (1, 2).

The cardiac stromal compartment consists of a great variety of cells, including mesenchymal and immune cells (3). They hold many roles in the heart's physiology, such as paracrine signals, extracellular matrix production (4), and affect the mechanical properties of the tissue (5–7). Conversely, the cardiac stromal compartment is affected itself by multiple pathological conditions (7–10), thus playing a key role in the pathophysiological response of the heart to different insults.

With the increasing incidence of cardiac diseases and the search of new therapeutic targets, better understanding the role of the stroma as the mediator of cardiac homeostasis versus disease initiation and progression, is of paramount importance as it may ultimately unveil significant pathways to be exploited or targeted for translational purposes.

The stromal compartment represents a non-classical player in this scenario, and this Research Topic has gathered contributions focusing on stromal cells, such as cardiac fibroblasts and resident macrophages, in cardiac homeostasis and disease establishment and progression. The scientific works presented depict a complex and multifaceted scenario, where new signaling pathways triggering fibrosis and remodelling, and the connection between cardiac and immune cells are central.

Rui et al. have investigated the molecular mechanisms of cardiac fibrosis, which is the main pathogenetic trigger in adverse cardiac remodeling, leading to negative prognosis in heart disease patients. They studied the modulation of SPARC-related modular calcium-binding protein 2 (SMOC2), a secreted modular protein regulating cell-matrix interactions, in cardiac fibrosis models both *in vitro* and *in vivo*. AAV9-mediated shRNA SMOC2-knockdown partially reverted the phenotype in a mouse model of cardiac fibrosis mediated by isoproterenol infusion. Moreover, SMOC2 suppression opposed myofibroblast differentiation of TGFβ-treated neonatal cardiac fibroblasts through inhibition of the ILK/p38 signaling, suggesting a novel preventative strategy for the management of cardiac remodeling.

On the role of inflammation and the crosstalk between cardiac and immune cells, Papotti et al. investigated whether macrophage polarization in epicardial and pericardial adipose tissue depots may correlate with circulating stress and/or clinical markers in patients with chronic coronary heart disease. The authors found evidence in these patients of increased inflammatory reprogramming of resident macrophages in cardiac fat depots, compared to subcutaneous fat, particularly for epicardial fat which is known to share the microcirculation with the myocardium. Interestingly, dyslipidemic patients showed significantly higher NOS2 expression in adipose tissues. These results suggest that coronary heart disease may be linked to an altered macrophage polarization in cardiac-associated fat depots, especially in epicardial adipose tissue, possibly representing both a pathophysiological mechanism and a therapeutic target for cardiovascular inflammation.

Acute myocardial infarction (MI) triggers an inflammatory response and its intensity is associated with the consequent development of heart failure (HF) (11). Anti-inflammatory therapies post-MI are available, but their efficacy still remains controversial. Wang et al. have reported a whole-genome expression analysis in peripheral blood mononuclear cells (PBMCs) from MI patients, searching for dynamic immune cell profiles. Results showed enriched pathways involved in inflammation and metabolic reprogramming, and that PBMCs from the HF subgroup were characterized by persistent upregulation of inflammation and coagulation genes. These results shed new light on the heterogeneity of PBMCs in HF patients, supporting the mechanistic involvement of immune cells in post-MI inflammation and its cardiac sequelae. Hence, personalized anti-inflammatory therapies should be considered for efficient clinical management of ischemic patients.

Following up with post-MI inflammation, Wilmes et al. studied the histological expression patterns of iNOS and nitrotyrosine (as a metabolite marker of NO production) in post-mortem human infarcted hearts. iNOS expression is considered a macrophage infiltration and activation marker that can contribute to post-ischemic oxidative stress. Results showed increased iNOS expression in resident macrophages in infarcted hearts. Moreover, histological proximity analysis evidenced how nitrotyrosine staining in tissue sections peaked within 10–15 µm from iNOS-positive cells, suggesting a significant iNOS-dependent contribution to oxidative stress by resident macrophages in the post-ischemic myocardium.

Gene expression signatures pointing to the relevance of the stromal cell compartment have come also from systematic analyses. Atrial fibrillation is the most frequent form of arrhythmia, with increased risk of major adverse cardiovascular events. Liu et al. explored omic profiles in atrial fibrillation by weighted gene co-expression network analysis (WGCNA) in a transcriptomic dataset of 10 atrial tissue samples from patients with permanent fibrillation versus 20 controls, to identify novel key genes involved the aging-related molecular profile of the disease. Their results identified several hub genes with diagnostic power, and the functional enrichment analysis highlighted their association with multiple pathways of interest, such as calcium trafficking, cAMP and PPAR signaling, and TGF-β signaling in cardiac fibrosis. Thus, they proposed novel potential biomarkers and therapeutic targets for atrial fibrillation in the aging population, which lay in the stromal compartment of the heart.

A peculiar contribution came form Akbar et al. who performed a systematic review of *in vivo* studies assessing the anti-apoptotic and anti-fibrotic effects of exercise training in animal models of hypertensive heart. Exercise is a well-acknowledged good habit for the prevention of cardiovascular diseases, but the key molecular mechanisms behind this beneficial effect are not clear yet. From the final 11 studies included, the authors highlighted increased cardiac cell survival associated to physical exercise through several pathways, such as IGF-1, PI3K, and Akt, and reduced activation of apoptotic pathways, including Bad and Bax. Studies also showed amelioration of physiological features of left ventricle fibrosis with reduced activation of MAPK, p38, and PTEN with exercise training. Overall, this review highlights the important preventive role of exercise on apoptotic and fibrotic pathways in the heart under hypertensive insult, evidencing how the cardiac stromal compartment could also support the beneficial effects of training on the cardiac muscle.

In conclusion, cardiac diseases, whether acute or chronic, inherited or acquired, are often characterized by parenchymal cell loss, inflammation, fibrosis, and altered mechanical properties of the tissue. In this scenario, the stromal compartment plays a key role in the development of the disease which is increasingly recognized. Understanding better the role of cardiac stromal cells in both physiological and pathological conditions is of paramount importance in the development of new therapeutic approaches. Many challenges still exist, but scientists in this interdisciplinary research field look forward to future exciting developments.
